# Acyclovir as a Novel Treatment for Severe Chronic Active Epstein-Barr Virus

**DOI:** 10.7759/cureus.62070

**Published:** 2024-06-10

**Authors:** Mary Therese Thomas, Philip Mardock, Kedareeshwar S Arukala

**Affiliations:** 1 Internal Medicine, Grand Strand Medical Center, Myrtle Beach, USA

**Keywords:** epstein-barr virus, use of antibiotic, antiviral therapy, chronic active epstein-barr virus (caebv), new therapy approaches, ‏acyclovir, ebv hepatitis

## Abstract

Epstein-Barr virus (EBV) is a widely infectious pathogen affecting most of the global population at some point in their life. While, typically, primary infections are subclinical, chronic persistence of the virus due to T-cell proliferation can cause severe complications. Acute hepatitis due to chronic active EBV (CAEBV) has rarely been documented. This case details a previously healthy 81-year-old woman who presented with complaints of diffuse abdominal pain, nausea, and vomiting. Her diagnostic workup demonstrated an EBV infection with worsening thrombocytopenia, transaminitis, and hepatocellular liver injury with acute ascites. Her hospitalization was resistant to the traditional supportive treatment of EBV, requiring intensive care management and unorthodox therapy. Although antivirals have demonstrated limited utility in the treatment of CAEBV, the severity of her illness and refractory hospital course necessitated the use of acyclovir. She made a complete recovery with no deficits. The case demonstrates the presentation of acute hepatitis and ascites as a result of CAEBV, the clinical sequelae, and acyclovir as a potential new treatment option.

## Introduction

Epstein-Barr virus (EBV), also known as human herpesvirus 4 (HHV4), is a highly infectious pathogen that affects 95% of the world’s population at some point in their life [1]. Infection with EBV can result in infectious mononucleosis (IM), colloquially known as “the kissing disease” or “mono,” named for its transmission through saliva. IM is the primary infection of EBV and is a self-limiting process characterized by low-grade fever, sore throat, and lymphadenopathy [2]. Although many may recognize the name, it is more common to be infected by the virus and be asymptomatic than present with the clinical triad. While it is a relatively new area of study, EBV is unique among the herpes viruses in that it can persist in its host indefinitely and lead to many long-term complications. These infections are called chronic active EBV (CAEBV) [1]. IM-like symptoms can be present in CAEBV, but additional involvement of virtually any organ system is possible due to the proliferation of T-cells. The most common manifestations of CAEBV are oncologic complications, such as B and T-cell lymphomas [1]. Cases previously published about CAEBV show the virus masquerading as pneumonia, myocarditis, and pancreatitis [3]. Some reports have seen manifestations of neurologic syndromes such as Guillain-Barre syndrome, transverse myelitis, meningitis, and peripheral or optic neuritis [4].

Among these case reports, there are very few cases of EBV and its associated hepatic T-cell infiltration resulting in acute hepatitis with jaundice and ascites. The first documented case of these symptoms without other signs of hepatic dysfunction was published as recently as 1999 [5]. Less than 10% of young adults and 30% of elderly patients have hepatic involvement, with less than 5% having further complications such as jaundice [6,7]. The number of EBV-associated oncologic deaths continues to rise. The Global Burden of Disease (GBD) study of EBV estimated that 17% of lymphoproliferative deaths between 1990 and 2017 can be attributed to the virus [8].

Despite its global effect, the diagnostic criteria of CAEBV have yet to be well defined. IgM and IgG antibodies directed against viral capsid antigens confirm the diagnosis of IM or an acute infection [9]. IgG antibodies to antiviral capsid antigen (VCA) and Epstein-Barr nuclear antigen (EBNA) will be present for life after initial infection, so finding a unique marker to identify CAEBV is challenging. The following case of hepatitis secondary to severe CAEBV results in a favorable outcome for the patient and perhaps can serve as a model for future treatment options given the use of acyclovir as an unconventional treatment.

## Case presentation

An 81-year-old female presented to the hospital complaining of diffuse abdominal pain, nausea, and vomiting for four days. Her prior medical history included irritable bowel syndrome, unspecified chronically low sodium, generalized anxiety disorder, and non-Hodgkin's lymphoma in remission for several years.

On her initial evaluation, the patient reported several episodes of vomiting without hematemesis. She denied fever, chills, fatigue, sore throat, recent illnesses, or sick contacts. Her physical exam demonstrated diffuse abdominal pain, most notable at the epigastrium, accompanied by distension with active bowel sounds. Her vitals were stable, with initial readings showing a temporal temperature of 96.8° Fahrenheit, respiratory rate of 17, heart rate of 77, and blood pressure of 175/79. The patient's labs were notable for low sodium and chloride at 128 mmol/L and 87 mmol/L, respectively. Per the patient, these levels were considered her baseline. Her hepatic panel demonstrated mild elevation of her aspartate aminotransferase (AST), alanine transaminase (ALT), and alkaline phosphatase (ALP) (Table 1). The rest of her metabolic panel and blood cell counts were within normal limits. A lipase level obtained was within normal limits. A urine analysis showed 15 mg/dL ketones and 1+ protein.

**Table 1:** Trend of liver enzymes and platelets throughout the patient's hospital stay. Acyclovir administration occurred on day six (bold).

Time	Aspartate aminotransferase	Alanine transaminase	Alkaline phosphatase	Platelets
	15-46 U/mL	13-369 U/mL	38-126 U/mL	156-352 K/mm3
Day 1	61	68	95	223
Day 2	102	96	88	186
Day 3	248	266	140	148
Day 4	391	457	193	72
Day 5	501	535	165	23
Day 6	703	911	197	20
Day 7	741	936	192	30
Day 8	706	924	208	34
Day 9	540	741	255	48
Day 10	352	587	249	67
Day 11	327	565	322	77
Day 12	357	538	384	82
Day 13	232	493	404	87

An initial computed tomography (CT) with contrast of the abdomen/pelvis revealed subtle inflammation in the superior, most central, and posterior abdomen surrounding the right adrenal gland and the portocaval region. The radiologist expounded on these findings, suggesting possible duodenitis or acute pancreatitis. The working diagnosis with this initial evaluation included duodenitis/gastritis with an irritable bowel flare. The initial workup included stool antigen testing for *Helicobacter pylori*, hepatitis profile, urine drug screen, acetaminophen levels, and qualitative human immunodeficiency virus antigen/antibody (HIV), resulting in negative findings.

**Figure 1:**
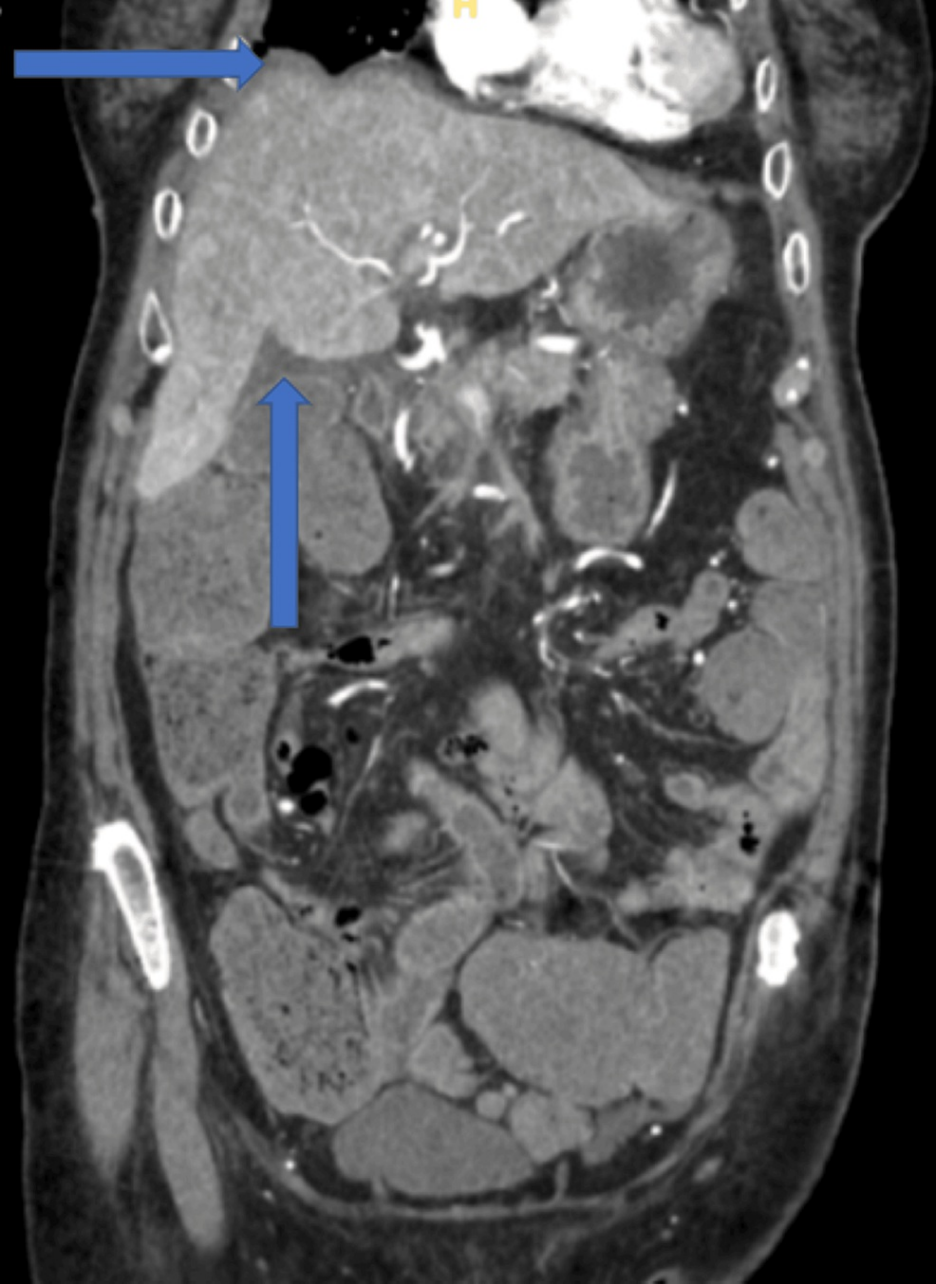
Computed tomography angiography of the abdomen/pelvis (coronal view). Radiologic impression was suggestive of diffuse, multifocal gastroenteric colitis with new ascites and heterogeneous hepatic perfusion.

The patient's bowel regimen successfully elicited a bowel movement on day two of admission but was complicated by gross hematochezia and worsening abdominal pain. A repeat CT angiography was ordered due to concerns of mesenteric ischemia. It demonstrated no ischemia but did show the development of diffuse, multifocal gastroenteric colitis with new ascites and heterogeneous hepatic perfusion (Figure 1). In addition, the patient's lab work showed worsening thrombocytopenia and transaminitis (Table 1). Her total bilirubin was elevated to 5.1 u/L with a direct bilirubin of 0.1 u/L. Her sodium levels continued down-trending to a low of 114 mmol/L. The patient was started on antibiotics, including intravenous (IV) levofloxacin 750 mg daily and IV metronidazole 500 mg. An abdominal ultrasound demonstrated normal liver echogenicity without signs of cholecystic involvement and patent venous flow. Additional lab workup was ordered, including coagulation studies of prothrombin time (PT)/international normalized ratio (INR), lactate dehydrogenase (LDH), and fibrinogen, which revealed an elevation of LDH to 847 u/L and a D-dimer level greater than 7000. PT/INR was less than 1.5, and fibrinogen was within normal limits.

A steady decline in platelets and sodium further complicated the patient's continued transaminitis and gastroenteric colitis over the next several days. Her platelets declined to a low of 23,000/µL, and sodium ranged from 114 mmol/L to 118 mmol/L. A peripheral smear showed thrombocytopenia with giant forms but no concerns of hemolysis. Hematology was consulted, and concerns were raised that the patient's thrombocytopenia was most likely an infection-triggered process. She was started on 40 mg of IV methylprednisolone every eight hours. Her low sodium was accompanied by an encephalopathy displayed as somnolence, albeit with retained orientation. She was urgently transferred to the intensive care unit (ICU) to begin an infusion of 3% hypertonic saline.

This therapy improved the patient's low sodium, but she continued to demonstrate recalcitrant thrombocytopenia and steadily rising transaminases with LDH greater than 1000 u/L by day six of her admission (Table 1). Upon review of the ordered studies and labs, autoimmune hepatitis was ruled out with immunoglobulin factors and antineutrophilic cytoplasmic antibody testing. A negative urinary drug screen and acetaminophen levels ruled out toxic liver injury. HIV and typical viral hepatitis were ruled out early in the patient's admission. However, the patient's titers for EBV were significantly elevated despite a lack of possible sick contacts or other possible exposures (Table 2).

**Table 2:** EBV titer levels. EBV: Epstein-Barr virus; Ag: antigen; Ab: antibody.

	Patient values	Normal range
EBV capsid Ag IgG Ab	436	0.0-17.9 U/mL
EBV capsid Ag IgM Ab	>160.0	0.0-35.9 U/mL
EBV nuclear Ag Ab	345	0.0-17.9 U/mL

A literature review at that time revealed that, albeit rare, CAEBV can result in hepatitis and ascites without other liver dysfunction. The patient received multiple treatment modalities to attempt to correct her thrombocytopenia, low sodium, and hepatitis but continued to remain in a critically ill state. IV acyclovir 500 mg every eight hours was initiated as an experimental therapy on hospital day six.

**Figure 2:**
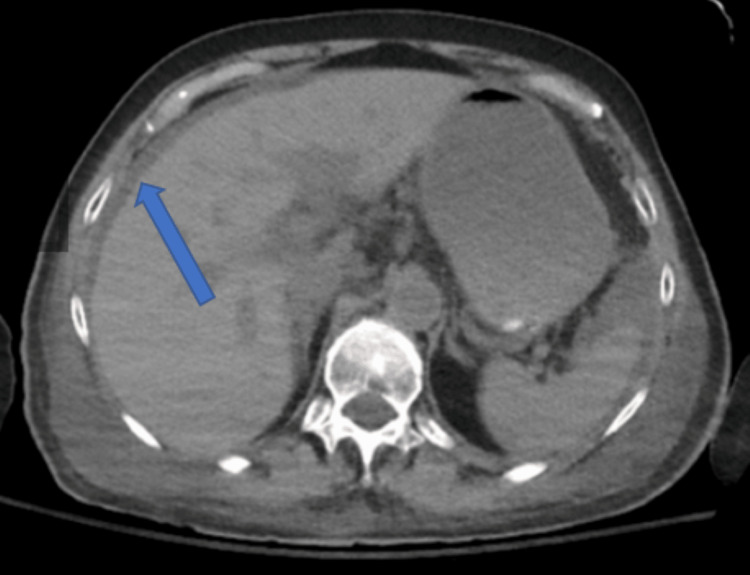
CT without contrast of the abdomen/pelvis (transverse view). Radiologic impression read as residual moderate intra-abdominal ascites.

**Figure 3:**
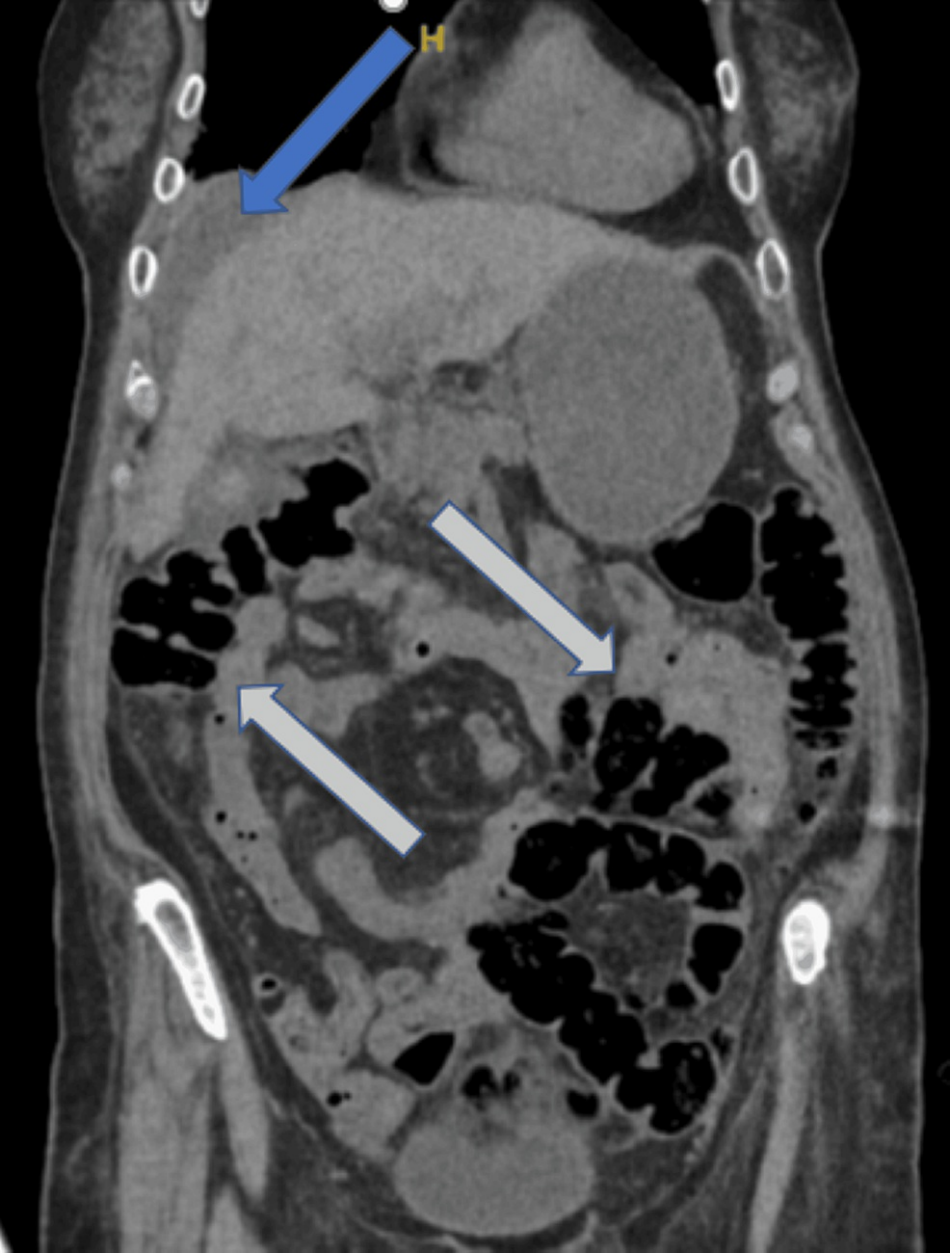
CT without contrast of the abdomen/pelvis (coronal view). Radiologic impression read as residual moderate intra-abdominal ascites.

The patient drastically improved after just one day of the antiviral therapy with improvement of her encephalopathy and subjective report of strength. Her transaminases steadily downtrended and LDH returned to within normal limits. In addition, her hyponatremia returned to her baseline and her abdominal pain ceased altogether. After seven days of IV therapy, she was discharged home on oral acyclovir 800 mg twice daily for seven additional days, for a total therapy duration of 14 days. She had no complications at the time of discharge besides a moderate amount of residual ascites (Figures 2, 3).

## Discussion

While this case demonstrates a rare and understudied complication of CAEBV, it is among the first to show a reduction in the severity of symptoms using acyclovir as adjunctive therapy to corticosteroids in latent disease after steroid monotherapy was unsuccessful. Treatment of acute EBV is typically supportive, and viral syndrome is self-limited. Agents, including antivirals, immunosuppressants, and other more radical therapies, such as plasmapheresis, have been proven non-superior to traditional supportive therapy [10].

Latent disease is characterized by a high EBV DNA load (103-107 copies/mL) [10]. Although this patient never had her DNA load measured, her serological profile showed an IgG antibody (Ab) at 436 U/mL, IgM Ab > 160 U/mL, and nuclear antigen Ab at 345 U/mL, consistent with a previous infection or reactivation of the virus. Our patient was incidentally found to have the disease after ruling out all other diagnoses and searching for the cause of her acute hepatitis. She had no known exposure to the virus and did not knowingly have the disease recently or in her youth. What is interesting to note is that before hospitalization, she did have a pre-existing diagnosis of lymphoma. This detail brings to question whether her CAEBV had been causing complications long before this hospitalization.

A minimal amount of literature supports using antivirals as therapy, either alone or adjunctive, in CAEBV infections. Acyclovir works by inhibiting viral DNA synthesis and competes for transcription into viral DNA, disrupting replication [11]. Without active replication, its mechanism of action would not be particularly effective in latent infections. Acyclovir has been proven to help reduce viral shedding in the oropharynx and points toward non-statistically significant clinical improvement in most literature reviews [11]. Unfortunately, treatment dosage and duration are highly variable amongst the reported case studies, making accurate comparison difficult [12]. Many call for more research on the topic and its use in latent EBV, but more extensive studies or reviews have yet to be initiated [13].

This report illuminates one of the severe complications of EBV and demonstrates improvement with the utilization of acyclovir to decrease the duration of symptoms and resolve acute hepatitis. By sharing this case, we hope to shed light on often overlooked complications and a potential treatment to improve clinical outcomes for patients with EBV and this rare presentation.

## Conclusions

When acute hepatitis occurs in patients without apparent cause, it is essential to rule out CAEBV, a well-known virus to the general population that may become latent and present with severe complications such as lymphadenopathy, pneumonia, hematologic abnormalities, or less commonly, central nervous system dysfunction and hepatitis. While treatment for active disease is supportive, acyclovir and other antivirals remain an avenue of treatment that has been understudied as adjunctive therapy with steroids to reduce the severity of symptoms and duration of illness.
